# Iron Ladies – How Desiccated Asexual Rotifer *Adineta vaga* Deal With X-Rays and Heavy Ions?

**DOI:** 10.3389/fmicb.2020.01792

**Published:** 2020-07-31

**Authors:** Boris Hespeels, Sébastien Penninckx, Valérie Cornet, Lucie Bruneau, Cécile Bopp, Véronique Baumlé, Baptiste Redivo, Anne-Catherine Heuskin, Ralf Moeller, Akira Fujimori, Stephane Lucas, Karine Van Doninck

**Affiliations:** ^1^Research Unit in Environmental and Evolutionary Biology (URBE), Laboratory of Evolutionary Genetics and Ecology (LEGE), NAmur Research Institute for Life Sciences (NARILIS), University of Namur, Namur, Belgium; ^2^Research Unit in Environmental and Evolutionary Biology (URBE), Institute of Life, Earth & Environment (ILEE), University of Namur, Namur, Belgium; ^3^Laboratory of Analysis by Nuclear Reaction (LARN), NAmur Research Institute for Life Sciences (NARILIS), University of Namur, Namur, Belgium; ^4^Space Microbiology Research Group, Radiation Biology Department, Institute of Aerospace Medicine, German Aerospace Center (DLR), Cologne, Germany; ^5^Department of Natural Sciences, University of Applied Sciences Bonn-Rhein-Sieg (BRSU), Rheinbach, Germany; ^6^Department of Basic Medical Sciences for Radiation Damages, National Institute of Radiological Sciences (NIRS), Chiba, Japan

**Keywords:** bdelloid rotifer, LET, DNA damage, extremophile, astrobiology, panspermia

## Abstract

Space exposure experiments from the last 15 years have unexpectedly shown that several terrestrial organisms, including some multi-cellular species, are able to survive in open space without protection. The robustness of bdelloid rotifers suggests that these tiny creatures can possibly be added to the still restricted list of animals that can deal with the exposure to harsh condition of space. Bdelloids are one of the smallest animals on Earth. Living all over the world, mostly in semi-terrestrial environments, they appear to be extremely stress tolerant. Their desiccation tolerance at any stage of their life cycle is known to confer tolerance to a variety of stresses including high doses of radiation and freezing. In addition, they constitute a major scandal in evolutionary biology due to the putative absence of sexual reproduction for at least 60 million years. *Adineta vaga*, with its unique characteristics and a draft genome available, was selected by ESA (European Space Agency) as a model system to study extreme resistance of organisms exposed to space environment. In this manuscript, we documented the resistance of desiccated *A. vaga* individuals exposed to increasing doses of X-ray, protons and Fe ions. Consequences of exposure to different sources of radiation were investigated in regard to the cellular type including somatic (survival assay) and germinal cells (fertility assay). Then, the capacity of *A. vaga* individuals to repair DNA DSB induced by different source of radiation was investigated. Bdelloid rotifers represent a promising model in order to investigate damage induced by high or low LET radiation. The possibility of exposure both on hydrated or desiccated specimens may help to decipher contribution of direct and indirect radiation damage on biological processes. Results achieved through this study consolidate our knowledge about the radioresistance of *A. vaga* and improve our capacity to compare extreme resistance against radiation among living organisms including metazoan.

## Introduction

The exposition of cells to ionizing radiations (IR) generates a succession of physical, chemical and biological processes that differ in time, spatial and energy-scale leading to a cellular response complicated to predict (Joiner and van der Kogel, 2018). The ionization density in particle tracks is usually characterized by a physical parameter called Linear Energy Transfer (LET) describing the average energy (in keV) given up by a charged particle traversing a distance of 1 μm. High-Z charged particles (such as Fe ions) are high LET particles that will produce dense ionizations along their path while photons (like X-rays) which produce sparse ionizations are considered as low LET IR (Joiner and van der Kogel, 2018; Hagiwara et al., 2019). Although a same dose of radiation leads to the same amount of ionizations, the differences in their spatial distribution is translated into different types of damages at biological level. It was demonstrated that sparse ionizations originating from low LET radiations mainly induce DNA base oxidation and single strand breaks (Lehnert, 2007). Contrastingly, complex DNA damages including clustered double strand breaks (DSBs) are preferentially created following high-LET exposition resulting in a poor survival of irradiated organisms (Semenenko and Stewart, 2004). Moreover, an increasing amount of studies highlighted that the complexity of damages induced by high LET particles was associated to lower repair kinetic leading to an increase in chromosomal aberrations and cancer incidence (Loucas and Cornforth, 2013; Penninckx et al., 2019). Although the health risks from exposure to terrestrial radiations (electrons and photons) are well known, the ones associated to heavy ions particles (HZE) remain poorly understood since they are only naturally present in space. Therefore, the lack of data regarding whole-body response following HZE exposure and solutions to counteract their deleterious effects were identified as major showstoppers for future long-duration space missions (White and Averner, 2001; Cucinotta and Durante, 2006). Within the animals, bdelloid rotifers are known to survive high doses of high and low LET ionizing radiation (Gladyshev and Meselson, 2008; Hespeels et al., 2014; Jönsson and Wojcik, 2017), making them a new, suitable model system for space research.

Bdelloid rotifers are among the smallest animals on Earth, with most species being less than 1 mm in size and containing ∼1,000 cells. Despite their small size, these eutelic (i.e., a fixed number of cells at maturity) metazoans have a complete nervous, muscular, digestive and excretory system (Ricci and Melone, 2000). One remarkable feature of bdelloid rotifers, being even more unusual among animals, is the absence of males, vestigial male structures or hermaphrodites in any of the populations studied within the 460 described morphospecies (Segers, 2007). Bdelloid rotifers are females apparently cloning themselves since millions of years. While the exact nature of their parthenogenetic reproductive mode remains unknown, bdelloid rotifers are considered an “evolutionary scandal” because they persist without sexual reproduction (Maynard Smith, 1986).

Living all over the world, mostly in semi-terrestrial environments, they appear to be extremely stress tolerant. Around 90% of bdelloid species were described to live in semi-terrestrial habitats and in particular limno-terrestrial environments such as mosses and lichens (Melone and Fontaneto, 2005). Their desiccation tolerance at any stage of their life cycle allows bdelloid rotifers to thrive in semi-terrestrial environments but also confers tolerance to a variety of stresses including freezing, deep vacuum, UV and high doses of ionizing radiation (Ricci et al., 2005; Gladyshev and Meselson, 2008; Fischer et al., 2013; Hespeels et al., 2014; Jönsson and Wojcik, 2017; Ricci, 2017).

Nevertheless, an overview of the radiation tolerance among bdelloids is lacking and only few radiation studies have been published. No lethal dose is currently available in the literature. In 2008, Gladyshev and Meselson demonstrated that two bdelloid rotifer species maintained hydrated, *Adineta vaga* and *Philodina roseola*, are extraordinary resistant to ionizing radiation (IR), surviving up to 1,200 Gy of gamma radiation with fecundity and fertility showing a dose response (Gladyshev and Meselson, 2008). These doses were contrasting the Lethal Dose 50 (i.e., dose required to kill 50% of the irradiated population) of mammalian cells ranging from 2 to 6 Gy after X-ray exposure (Kohn and Kallman, 1957; Mole, 1984). In 2014, Hespeels et al. demonstrated that the rehydration process of desiccated *A. vaga* individuals was not affected by doses up to 800 Gy of protons 1,7 MeV. In 2017, Jönsson and Wojcik highlighted that desiccated bdelloid *Mniobia russeola* is able to tolerate exposure to iron ions up to at least 2,000 Gy with no apparent impact in term of survival and egg production (Jönsson and Wojcik, 2017).

By analogy with the desiccation- and radiation resistant bacterium *D. radiodurans* (Mattimore and Battista, 1996), it has been postulated that the extraordinary radiation resistance of bdelloid rotifers reflects an adaptation to survive desiccation and a high capacity to deal with numerous DNA DSBs (Gladyshev and Meselson, 2008; Gladyshev and Arkhipova, 2010). Indeed, high radiation doses are known to induce numerous cellular damages, through the formation of reactive oxygen species (ROS) and inducing DNA DSBs (Riley, 1994). We previously demonstrated that *A. vaga* accumulate DNA double strand breaks (DSBs) with time spent in dehydrated state. Upon rehydration, rehydrated animals were able to reassemble large fragments of DNA supporting the presence of active DNA repair mechanisms. Even when the genome was entirely shattered into small DNA fragments by exposure to 800 Gy of protons 1.7 MeV radiations, *A. vaga* individuals were able to efficiently recover from desiccation and to repair the DNA DSBs (Hespeels et al., 2014). Krisko et al. (2012) demonstrated that *A. vaga* is far more resistant to IR-induced protein carbonylation than is the much more radiosensitive nematode *Caenorhabditis elegans*. Therefore, like bacteria *Deinococcus radiodurans*, bdelloid rotifers appear to survive such extreme conditions because of efficient antioxidants and DNA repair mechanisms (Flot et al., 2013; Latta et al., 2019). However, the exact biological processes of these resistance mechanisms remain largely unknown.

Space exposure experiments from the last 15 years have unexpectedly shown that several terrestrial organisms, including some multi-cellular species, are able to survive in open space without protection. In the absence of protective effects from the atmosphere and the magnetic field, organisms are exposed to a mixture of radiations and accelerated particles. More specifically, biological samples in space are expected to be exposed to galactic cosmic rays (GCR) and to radiation bursts generated by solar eruption (Simpson, 1983; Wilson, 1991). GCR, originating from extra solar events like supernova, are composed of 83% protons, 12% alpha particles and 1.5% high atomic number and energy particle (HZE) including Fe (Ferrari and Szuszkiewicz, 2009). Despite reduced occurrence in terms of particle number, Fe ions driven by their huge LET are the primary component of GCR in term of energy deposited on the target (Leuko and Rettberg, 2017; Moeller et al., 2017). In addition, the collision of GCR with matter may result in the generation of low LET gamma and X-ray radiation (Dartnell, 2011; Leuko and Rettberg, 2017). The robustness of bdelloid rotifers toward IR suggests that these tiny creatures can possibly be added to the still restricted list of animals used for astrobiology experiments, including exposure to simulated deep space environment. Bdelloid rotifers can indeed contribute significantly to our understanding of living in extreme environments. The bdelloid species *A. vaga*, whose genome was sequenced (Flot et al., 2013), got recently selected for an astrobiology experiment aimed at identifying and quantifying biological damage suffered by bdelloids when exposed to space environment. Through this project, bdelloid rotifers will be exposed to a combination of full-spectrum electromagnetic radiation from the Sun, cosmic particle radiations, vacuum, wide temperature fluctuations and microgravity. In the preparation of this experiment, the present manuscript aims to determine the limits and consequences of bdelloid rotifer extreme resistance to radiation.

In this study, the resistance of *A. vaga* versus multiple sources of radiation was investigated. The biological response of these animals to high doses of low and high LET was evaluated, respectively, using X-ray, protons 4 MeV and Fe 0.5 GeV particles. The high resistance of *A. vaga* individuals to radiation prone them as a good model to study the prevalence of direct and indirect damages coming from high doses of ionizing radiation (>100 Gy). Here, hydrated and desiccated *A. vaga* individuals were exposed to increasing dose of X-ray, and only desiccated *A. vaga* to proton and Fe ions. For each radiation dose, we evaluated the survival and the capacity of irradiated animals to produce viable offspring. These data were confronted with the amount of DNA damage, evaluated by pulsed field gel electrophoresis (PFGE), and with the capacity of irradiated animals to repair, at least in somatic cells, DNA breaks induced by X-ray, proton and Fe radiation. Results achieved through this study consolidate our knowledge about the radioresistance of bdelloid rotifer *A. vaga* and improve our capacity to compare extreme resistance against low and high LET radiation among living organisms including metazoan.

## Materials and Methods

### Bdelloid Rotifer Cultures

Experiments were performed using isogenic *A. vaga* clones issued from a single individual from the laboratory of Matthew Meselson at Harvard University (Flot et al., 2013). The cultures were maintained hydrated in Petri dishes supplemented with natural spring water (Spa), in thermostatic chambers at 21°C, and fed with sterilized lettuce juice (*Lactuca sativa*). Bacterial proliferation and dirt accumulation were avoided by changing the water minimum twice a month.

### X-Ray Exposure

Desiccated and hydrated *A. vaga* individuals were irradiated using an X-ray irradiator PXi X-RAD 225 XL, with a dose rate of ∼7.8 Gy/min (19 mA, 225 kV, no filter) available at LARN (UNamur-Belgium). Irradiation time was calculated from this dose rate to obtain the different doses. All samples, including control samples, were put on a refrigerated water bag to mitigate heating from X-ray. For each dose, a minimum of three replicates were recorded from at least two independent exposure campaigns (except for 5,000 and 7,500 Gy where 3 replicates per dose were obtained during one exposure campaign). The exposure of hydrated animals was limited to 1,500 Gy. For hydrated samples, a total of 2,000 to 5,000 living and healthy animals stored in 2 mL of Spa water supplemented by 100 μL of sterile lettuce juice were exposed to X-ray in refrigerated multi-well plates. As animals were metabolically active during irradiation and because maximum dose rate was limited, it was decided to expose hydrated *A. vaga* individuals to a maximum dose of 1,500 Gy. For desiccated *A. vaga* samples individuals were dried for 1 day (see desiccation protocol below) before irradiation to X-rays doses ranging from 100 to 7,500 Gy.

### 4 MeV Proton Exposure

Only desiccated *A. vaga* were irradiated with a homogenous broad proton beam defocused over 1 cm^2^ area, produced by a 2 MV Tandem accelerator (High Voltage Engineering Europa). A thorough description of the experimental set-up and the irradiation procedure is given in Wéra et al. (2011). Briefly, 4 MeV pristine proton beam was extracted in air through a 1 μm silicon nitride window. The linear energy transfer (LET) at the sample location was computed using SRIM software (Ziegler et al., 2010), using two stacked layers representing the setup: 1 μm Si_3_N_4_ exit window and a small 3 mm air gap between the exit window and the sample dish. The energy of transmitted particles was averaged and the LET was obtained for this average proton energy. The irradiation chambers were placed vertically on a sample holder fixed at the end of the beamline. Rotifer samples were thus positioned perpendicularly to the incoming beam. Homogeneity was achieved by defocusing the beam and checked using a passivated implanted planar silicon detector moved along *x* and *y* directions. Dose rate was assessed every millimeter in a 1 cm^2^ surface with errors less than 5% in the cell sample region. Dataset generated for this study was acquired through 6 campaigns of irradiation. Between campaigns, the dose rate ranged from 42 to 134 Gy min^–1^. The particle flux was accordingly adjusted using the classic broad beam formula (Turner, 2007) as shown below (Eq. 1):

(1) $$\dot{D}=\frac{1.610^{-9}LET\emptyset }{\rho }$$

Here the density ρ is taken as 1 g cm^–3^ and Φ is the proton beam flux in part cm^–2^ s^–1^. For each dose, a minimum of 3 replicates were recorded from at least 2 independent campaigns.

The desiccated *A. vaga* individuals were exposed to 4 MeV proton, with a LET of 10 keV/μm, ranging from 100 to 10,000 Gy.

### Fe 500 MeV/n Exposure

Only desiccated *A. vaga* individuals were also irradiated with iron ions (Fe 500 MeV/n, with a LET of 200 keV/μm and a dose rate of 10 Gy/min). Desiccated *A. vaga* individuals were stored in 0.2 mL PCR tubes and sent to the Heavy Ion Medical Accelerator in Chiba (HIMAC) facility at the National Institute of Radiological Sciences (NIRS) in Chiba (Japan). Two campaigns of irradiations were performed in January 2018 and June 2018, respectively. During the first campaign, samples were exposed to 250, 500, 1,000, and 2,000 Gy. In the second campaign, desiccated *A. vaga* rotifers were exposed to 1,000, 2,000, and 3,000 Gy Fe ions. For each campaign a set of 4 replicates per dose was prepared. In parallel, two sets of controls were prepared for each exposure campaign. One set consisted of desiccated *A. vaga* individuals that were sent to HIMAC facility and back to Belgium without irradiation (Control transport). The second set consisted of desiccated *A. vaga* individuals stored dry at controlled temperature (15°C) in LEGE laboratory (UNamur) for the whole duration of the exposure experiment (from sample preparation, exposure to HIMAC, and travel back to Belgium). For each campaign, it was impossible to rehydrate the whole dataset of desiccated samples on the same day due to the workload and analysis required post rehydration. As a consequence, samples were rehydrated randomly after their return to Belgium. Therefore, samples from first campaign were desiccated for a minimum and a maximum duration of 30 to 37 days, respectively. For the second campaign, the total desiccation ranged between 39 and 48 days.

### Desiccation and Survival Rate

For desiccation experiments, we used the protocol previously described in Hespeels et al. (2014). Briefly, healthy cultures were shortly washed in filtered Spa water. Individuals were collected using quick vortex and transferred to a 50 mL Falcon tube for centrifugation. The pellet of concentrated *A. vaga* individuals was resuspended in 1.25 mL of Spa water and placed in the center of Petri dishes containing 30 mL of 3% LMP agarose (Invitrogen). Plates with approximately 15,000–45,000 *A. vaga* individuals are placed in a climatic chamber (WEKK 0028) for desiccation following the optimized desiccation protocol: (1) linear decrease of relative humidity from 70 to 55% for 17 h (temperature: 23°C), (2) linear decrease of relative humidity from 55 to 41% for 1 h (temperature: 23°C), and (3) maintenance of 41% RH and 23°C during the whole exposure period. After desiccation, dried bdelloid individuals were rehydrated using 15 mL Spa^®^ water and stored at 21°C for 48 h. Bdelloids were considered alive when they had fully recovered motility or when the mastax moved in contracted individuals. All living and dead animals were collected separately. Then, the survival rate was estimated manually by counting living and dead individuals under the binocular. When the number of individuals was higher than ∼750, the number of individuals was extrapolated by counting 6 times the number of living/dead animals observed in 2 μL taken from a homogenously mixed culture in a final volume of 2–5 mL. Data are available in Supplementary Data S1. In the absence of intermediary exposure doses between 5,000 and 7,500 Gy, it was not possible to compute an accurate LD50 for each radiation type.

### Fertility Assays

The reproductive capacity was defined as the ability for each *A. vaga* individual to lay eggs and to develop clonal populations after being desiccated and potentially irradiated. We tested the fertility of 1 day desiccated and irradiated individuals by randomly selecting and isolating a minimum of 60 successfully rehydrated individuals per condition; each isolated female was deposited in a well of a 12-well petri plate. Each well was filled with 2 mL of Spa water and 50 μL of sterilized lettuce juice. After 30 days, wells were observed under a binocular stereoscope checking for: (1) the presence of a population (minimum 2 adults and 1 egg per well), (2) the presence of only eggs that did not hatch (+eventually the single adult from the start defined as a sterile individual), and (3) the presence of only dead individual (s). When the number of survivors after irradiation was below 60 individuals, the number of isolated individuals lacking to arrive at this total was automatically associated with the category dead individual (s). This correction avoids the overweighting of certain values following the drastic reduction of survivors at dose equal or higher than 5,000 Gy (X-ray and proton only). Data are available in Supplementary Data S1. Based on these data, it was possible to evaluate the minimal dose required to sterilize 50% of the irradiated population. A similar approach to the evaluation of a standard LD_50_ was applied here to define the Sterilizing Dose 50 (SD_50_). All curve fittings were performed with the OriginLab^®^ software (MA, United States). A dose-response equation was used to fit fertility data according to the following equation (Eq. 2):

(2) $$Sterilization=A_{1}+\frac{A_{2}-A_{1}}{1+10^{\left(logx0-x\right)p}}$$

Where A_1_ is the bottom asymptote (i.e., *representing the fertility of sterilized animals)*, A_2_ is the top asymptote (i.e., *representing the fertility of control animals)*, logx0 correspond to SD_50_ and p is the hill slope.

### Statistical Analysis

Data were expressed as means ± standard deviations (SD). Statistical analyses were performed using Rstudio software version 3.6.3 (RStudio Team, 2015) with packages car version 3.0–7 (Fox and Weisberg, 2019) and multcomp version 1.4–13 (Hothorn et al., 2008). Effects of radiation dose were analyzed using a Global Linear Model (using a Gaussian family). *Post hoc* comparisons at a 5% significant level were performed with General Linear Hypotheses (Tukey’s test) and multiple comparisons for GLM. This approach allowed not restricting the statistical analysis to the comparison of each condition *versus* the control condition (i.e., non-irradiated samples). Multiple comparisons between each dose ensure to discriminate significant differences between all conditions. Group characterized letters indicate the significant differences between groups: a significant difference (*P*-value < 0.05) between two conditions is observed when these conditions do not share any letter.

### Genomic DNA Integrity

The genomic integrity, i.e., the accumulation of DNA DSBs induced by desiccation or radiation, and the DNA repair kinetics were screened using Pulsed Field Gel Electrophoresis (PFGE) according to a protocol adapted from Hespeels et al. (2014). The genomic integrity was checked on hydrated and desiccated samples after their exposure to increasing dose of radiation. In order to screen the potential DNA repair process occurring post irradiation (and post rehydration for desiccated samples), the genomic integrity was evaluated after 2, 30, 8, 24, 48, and 168 h of rehydration by PFGE.

For each time point, *A. vaga* individuals were isolated in 1,300 μL of EDTA 50 mM pH 8 and Tris HCl 10 mM pH 8 and stored at −80°C until their analysis. Plugs were prepared by mixing a volume of 31 μL of EDTA 50 mM pH 8 and Tris HCl 10 mM pH 8 containing approx. 1,000 individuals and 19 μL of melted CleanCut agarose 2% (Bio-Rad, Hercules, CA, United States). After 15 min of polymerization at 4°C, plugs were individually transferred into 500 μL of digestion buffer 100 mM EDTA 50 mM Tris pH 8, supplemented with 1 mg/mL proteinase K (Thermo Fisher Scientific, Waltham, MA, United States) and 3.3% N-Lauroylsarcosine sodium solution. Plugs were stored 1 h at 4°C and incubated 18 h at 56°C. Then, plugs were rinsed three times with 0.5x Tris Borate EDTA (TBE) and kept for 3 h in 0.5x TBE at 4°C. Finally, plugs were stored at 4°C in 500 μL 0.5 M EDTA pH 8 after a last rinse with 0.5x TBE before use. Plugs were loaded in a 0.8% agarose gel (Lonza, Rockland, ME, United States) along *Saccharomyces cerevisiae* chromosomes as ladder (Bio-Rad, Hercules, CA, United States). Samples were run on PFGE using appropriate running program on BIORAD CHEF Mapper XA according to the following parameters: migration time = 22 h, temperature = 14°C, Volts/cm = 5.5, switch angle = 120°, switch times = 60 to 185 s with a linear ramp (resolution ranging from 225 to 1600 kb). To better separate DNA fragments within the shorter range (15–291 kbp) by PFGE, a similar protocol was followed except for a few changes: switch times = 1 to 25 s with a linear ramp for 24 h run time; temperature = 15°C; Volts/cm = 6; 1% agarose gel. Gels were stained with SYBR Gold (Invitrogen, Carlsbad, CA, United States) and analyzed by a BioRad Chemidoc XRS camera. Images were processed using ImageLab 3.0 and ImageJ. Photometric scans of gel lanes were obtained using the plot profile tool (ImageJ) calculated on straight lines of identical length.

## Results

### Evaluation of the Survival Rate of *A. vaga* Individuals Exposed to X-Ray, PROTONS and Fe Sources

Desiccated *A. vaga* individuals were exposed to increasing doses of ionizing radiation. The maximal doses achieved were about 3,000 Gy for Fe radiation, 7,500 Gy for X-ray and up to 10,000 Gy for protons 4 MeV. The survival rate of desiccated, irradiated individuals was investigated 2 days post rehydration.

**FIGURE 1:**
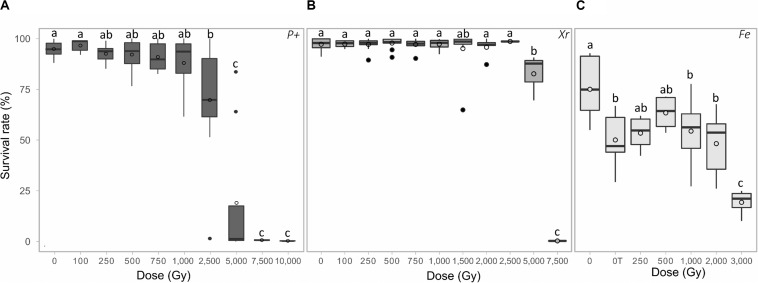
Survival rate of desiccated *A. vaga* individuals exposed to increasing doses of **(A)** protons (p+) **(B)** X-ray (Xr) **(C)** Fe. Survival rate of desiccated, irradiated *A. vaga* individuals was evaluated 48 h post rehydration. For Fe exposure, the dose of 0 Gy corresponded to laboratory control and “0T” corresponded to unirradiated, transported samples. X-ray and proton exposed samples were desiccated for a period of 24 h. Fe samples, irradiated in Japan, were stored approx. 1 month in desiccated state. A minimum of 3 replicates per dose was achieved. Data were visualized as boxplot (◯ = average; **–** = median). Letters indicate the significant differences between groups (*P*-value < 0.05).

For the proton exposure assay, unirradiated rotifers presented a survival rate of 94.8% (SD ± 3.5%). After exposure to protons 4 MeV, no significant differences were reported for samples exposed from 0 to 1,000 Gy (Figure 1A. group a). A significant decrease, in comparison with unirradiated samples, was reported after exposure to 2,500 Gy and more (Figure 1A. group b and c). Then, the survival rate of desiccated *A. vaga* individuals decreased to 19,1% (SD ± 34.1%) at 5,000 Gy, with a large variation observed between replicates. Finally, the clonal population of desiccated *A. vaga* individuals were dead when exposed to 7,500 Gy proton or more.

In comparison, X-ray exposed desiccated *A. vaga* do not show any significant decrease of survival rate until 2,500 Gy (Figure 1B. group a). Unirradiated samples were characterized by a survival rate of 95,6% (SD ± 2.5%). The survival rate at 5,000 Gy was 82,7% (SD ± 11.4%) and most animals (>99%) died after exposure to a dose of 7,500 Gy X-ray (see Figure 1B. group b and c, respectively).

Both for proton and X-ray exposure, few animals remained (<0.1%) active 2 days post rehydration at maximum doses (see Supplementary Data S1).

In the case of Fe irradiated dried samples, the survival of controls was affected by the transport to and from the irradiation site in Chiba (Japan). *A. vaga* individuals that were stored dry in our laboratory at 15°C for 1 month displayed a survival rate of 75.8% (SD ± 15.6) while unirradiated samples exposed to transportation were characterized by a survival rate of 50.1% (SD ± 13.3). No decrease of the survival rate was reported in *A. vaga* individuals exposed to Fe radiation up to 2,000 Gy (Figure 1C group b) in comparison with unirradiated, transported sample (Figure 1C “0T”). A significant decrease in survival rate of 30.8% was reported, in comparison with control samples, after an irradiation of 3,000 Gy to Fe (Figure 1C group c). The dose required to kill all the *A. vaga* individuals using Fe radiation was not achieved during this study.

**FIGURE 2:**
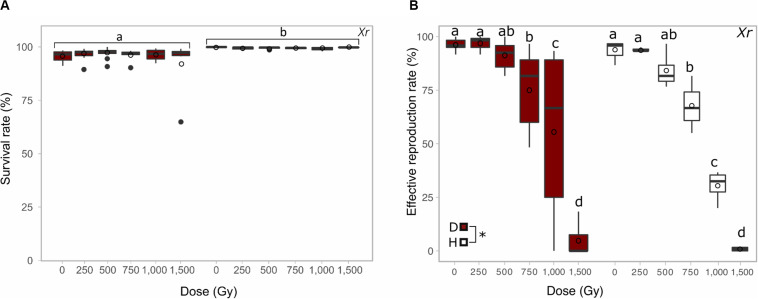
Comparison between the survival rate **(A)** and the reproductive capacity **(B)** of desiccated (red) and hydrated (white) *A. vaga* individuals exposed to X-ray. Survival rate was evaluated 2 days post rehydration or post radiation and evaluated on minimum 3 replicates. The reproductive capacity was evaluated with a minimum of 3 replicates per dose (see section “Materials and Methods”). For each replicate, reproduction was evaluated by direct observation under binocular 30 days after rehydration/radiation. Effective reproduction was validated when at least 2 adults and 1 egg per well were observed. Statistical analysis included comparison between and within each group of desiccated and hydrated individuals. Statistical analysis of survival data highlights the significant effect of hydrated/desiccated status. The reproductive capacity was characterized by independent effect of doses and hydrated/desiccated status. Data were visualized as boxplot (◯ = average; – = median). Letters or “*” indicate the significant differences between groups (*P*-value < 0.05).

The impact of irradiation on hydrated, active *A. vaga* animals was assessed up to 1,500 Gy using X-ray. No significant decrease of viability was reported in exposed animals up to the maximal dose of 1,500 Gy (Figure 2A group b). The significant difference in term of survival between hydrated and desiccated *A. vaga* individuals was attributed to the desiccation process that introduced more heterogeneity and mortality in the dataset of desiccated animals (Figure 2A group a). No sign of abnormal behavior was reported 2 days post irradiation.

### Comparing the Reproductive Capacity of *A. vaga* Individuals Exposed to X-Ray, Proton and Fe Sources

The reproductive capacity of irradiated *A. vaga* was evaluated by checking their capacity to lay eggs and to restart a new population of clonal *A. vaga* within a month post rehydration and isolation.

**FIGURE 3:**
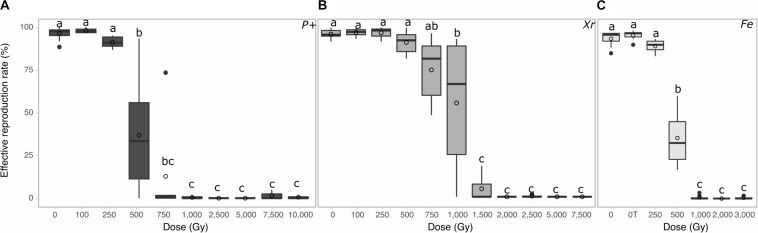
Reproductive capacity of desiccated *A. vaga* individuals exposed to **(A)** protons (P +) **(B)** X-ray (Xr) **(C)** Fe. The reproductive capacity was calculated with a minimum of 3 replicates per dose. For each replicate, 60 individuals were randomly isolated and individually placed in multi-well plates. Reproduction was evaluated by direct observation under binocular 30 days after rehydration/radiation. Effective reproduction was validated when at least 2 adults and 1 egg per well were observed. Data were visualized as boxplot (◯ = average; – = median). Letters indicate the significant differences between groups (*P*-value < 0.05).

For proton radiation, no significant decrease of fertility was reported up to 250 Gy (Figure 3A group a). However, the fertility started to decrease significantly at 500 Gy where a big variability between replicates was observed (Figure 3A group b). Above 500 Gy, most *A. vaga* individuals were sterilized by proton 4 MeV radiation (Figure 3A group c).

For X-ray radiation on desiccated samples, no significant decrease of fertility was reported at doses ranging from 0 to 750 Gy. However, a significant drop was reported at 1,000 Gy where a higher variability between replicates was observed. Then, a significant decrease of fertility was reported from 1,500 Gy, and most *A. vaga* individuals were sterilized at X-ray doses of 2,000 Gy and higher when desiccated (Figure 3B group c).

For Fe exposure, no significant difference in reproductive capacity between lab and transport control was reported. Moreover, no decrease of the fertility capacity was reported up to 250 Gy of Fe radiation (Figure 3C group a). A significant decrease of fertility was reported at 500 Gy (Figure 3C group b) and a complete sterilization of samples was reported at 1,000 Gy and higher (Figure 3C group c).

Based on collected data, it was possible to evaluate the minimal dose required to sterilize 50% of the irradiated *A. vaga* population. The median dose to sterilize a population [Sterilizing Dose 50 (SD_50_)] were 1,035 ± 20 Gy, 453 ± 23 Gy and 460 ± 1 Gy for X-ray, proton and iron radiation, respectively (see Supplementary Figure S1).

The failure of irradiated *A. vaga* individuals to restart a new population was not automatically linked with the non-ability of animals to produce eggs. Up to 2,500 Gy we noticed an increase of animals laying eggs that were unable to perform a complete embryonic development independently of radiation type (see Supplementary Figure S2). Multiple cellular divisions were reported in these sterile eggs that failed to reach a complete embryonic development (binocular observation of DAPI stained eggs, B. Hespeels and M. Terwagne, personal communication). Higher doses were characterized by an increased proportion of animals unable to lay eggs or dying within a month (see Supplementary Figure S2).

The effect of the hydration state (desiccated or hydrated) on irradiated animals was investigated through X-ray using doses ranging from 0 to 1,500 Gy. A higher impact of radiation on hydrated samples versus desiccated samples was highlighted by the comparison of both SD_50_. Those were evaluated to 1,125 ± 88 Gy and 889 ± 12 Gy for samples exposed desiccated or hydrated, respectively (see Supplementary Figure S1b). Statistical analysis (see section “Materials and Methods”) reveal the absence of interaction between factors dose and hydrated/desiccated status of samples when looking at the effective reproduction rate data. As suggested by SD_50_ a significant difference between hydrated and desiccated samples was reported: independently of the dose, the reproduction rate was significantly higher in desiccated samples than hydrated specimens. However, no significant difference was reported when data collected from hydrated and desiccated samples were compared for each tested dose (Figure 2B). At 1,000 Gy the fertility rate of desiccated samples was characterized by a high variability (55.5 ± 40.9%) in comparison with hydrated samples (30.4 ± 7.5%). 1% (SD ± 1%) of the exposed hydrated *A. vaga* were able to restart a population after exposure to 1,500 Gy X-ray in contrast to desiccated samples where 5% (SD ± 8%) of animals were still able to produce offspring after radiation. These differences were not statistically significant (Figure 2B). Nevertheless, a significant increase of animals unable to lay eggs or with premature deaths were reported in hydrated samples exposed to doses equal or higher to 1,000 Gy in comparison with desiccated, irradiated samples (Supplementary Figure S3b group c and d).

### Loss of Genomic Integrity in Desiccated and Irradiated *A. vaga* Individuals Exposed to Increasing Dose of Radiation

**FIGURE 4:**
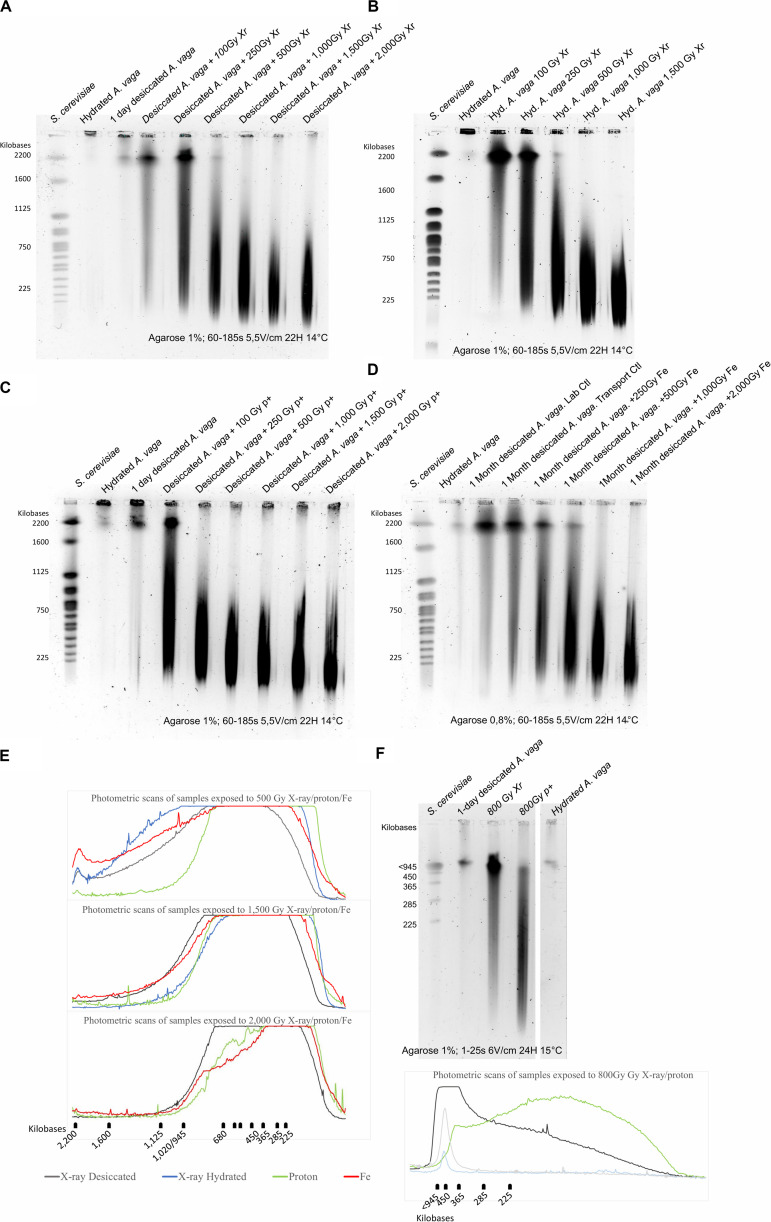
Loss of genomic integrity in *A. vaga* individuals exposed to increasing doses of X-ray **(A,B)**, Protons 4 MeV **(C)** and Fe 1 GeV **(D)**; Comparative analysis of photometric scans from samples exposed to 500; 1,500 and 2,000 Gy **(E)**; Comparative analysis of 1-day desiccated *A. vaga* individuals exposed to 800 Gy of X-ray and protons 4 MeV using short fragments run parameters (15–291 kbp) **(F)**. For X-ray exposure, rotifers were irradiated after 1 day of desiccation **(A)** or irradiated in hydrated state **(B)**. Rotifers were desiccated 1 day before exposure to Protons 4 MeV **(C)**. However, samples exposed to Fe were desiccated 1 month **(D)**. The first lane on the pulsed-field gel electrophoresis corresponds to the karyotype of Saccharomyces cerevisiae. Second lanes correspond to the control (1,000 hydrated *A.vaga* individuals). Third lanes of **(A,C)** correspond to DNA fragmentation induced by 1-day desiccation on 1,000 individuals. Other lanes correspond to 1,000 desiccated **(A,C,D)** or hydrated individuals **(B)** after exposition to 100, 250, 500, 1,000, 1,500 and 2,000 Gy (in exception of **(B)** where maximum dose was 1.500 Gy). For the comparative analysis focusing on short DNA fragments dose of 800 Gy of respectively X-ray and protons was used (Lane 3 and 4). Hydrated control was in lane 5. 1-day desiccated *A. vaga* individuals was at lane 2. The run parameters are documented under each gel. Photometric scans were generated using ImageJ (see “Materials and Methods” section). Top flat lines from photometric scans correspond to saturated levels.

To investigate how different types of radiation can affect the genomic integrity of *A. vaga* genome, the loss of genomic integrity induced by the accumulation of DNA DSBs was assessed using PFGE on 1,000 *A. vaga* individuals. As observed previously for *A. vaga* species (Hespeels et al., 2014), the genomic integrity of hydrated or 1 day desiccated bdelloid individuals was characterized by large DNA fragments remaining in the gel plug with a very weak signal around 2200 kbp (Figures 4A–C lanes 2 and 3; Figure 4B lane 2). At a dose of 100 Gy, loss of genomic integrity was observed through the visualization of a smear ranging between 225 to 2,200 kbp. Size of DNA fragments was negatively linked with the increase of the radiation exposure. After a dose of 1,000 Gy and higher, the genomes of *A. vaga* individuals were fragmented in DNA pieces mostly ranging between 225 and 1000 kbp (Figures 4A–D). Interestingly, for a similar dose, it was not possible to observe any significant variation in term of DNA fragmentation between X-ray, Proton and Iron radiation (see photometric scans Figure 4E). Moreover, when comparing hydrated and desiccated individuals exposed to similar dose of X-ray, no difference was reported (Figures 4A,B,E).

For samples exposed to Fe radiation, desiccated and unirradiated control presented a slight smear of DNA fragments ranging from small to high molecular weight. These controls were exposed to 1 month of dry storage and transported from Belgium to Chiba irradiation location and vice versa. These data are congruent with previous results suggesting that DNA DSB are accumulating with time in dried *A. vaga* even in absence of irradiation. As a consequence, the number of DNA DSBs may be overestimated in the dataset of samples exposed to increasing dose of Fe radiation.

Second, the genomic integrity of desiccated *A. vaga* individuals exposed to X-ray and proton was investigated with a resolution window of DNA fragments ranging between 15 to 242 kbp (Figure 4F). No smear was observed in hydrated and 1-day desiccated *A. vaga* individuals. However, both samples were characterized by a band of size >945 kbp. In contrast to previous results, different patterns between 1-day desiccated, irradiated *A. vaga* individuals exposed to 800 Gy of X-ray and proton were reported. The X-ray sample was characterized by intense signal for DNA fragments >600 kbp followed by a smear of decreasing intensity for DNA fragments <600 kbp. The sample exposed to proton radiation presented a reduced signal intensity for >600 kbp fragments and a smear of higher intensity than X-ray < 600 kbp. Similar patterns, highlighting progressive loss of genomic integrity, were reported when comparing 1-day desiccated samples irradiated at 500 and 1,000 Gy (see Supplementary Figure S4).

### Partial Restoration of Genomic Integrity After Exposure to Radiation

**FIGURE 5:**
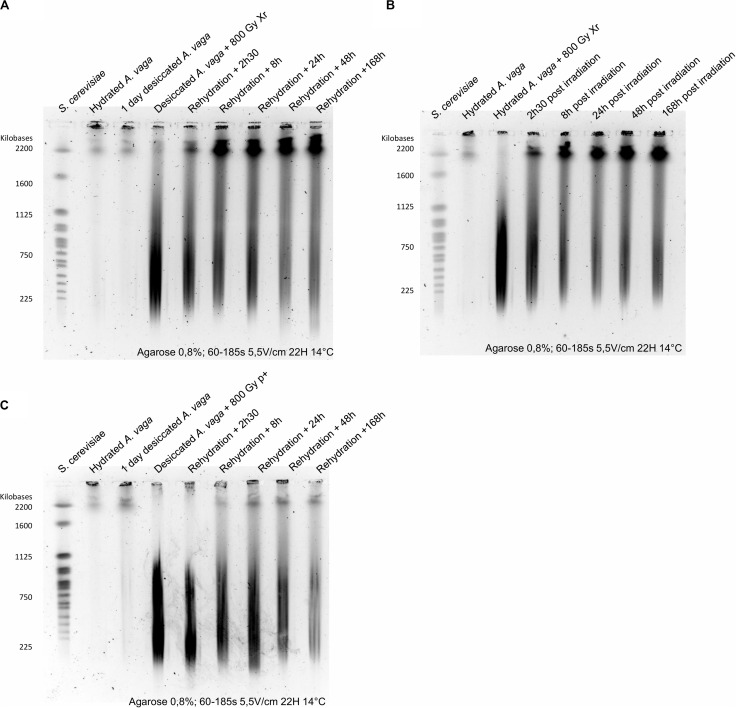
Repair kinetic of *A. vaga* individuals on hydrated or desiccated samples with exposure to 800 Gy of X-ray [**(A)** 1-day desiccated samples **(B)** hydrated animals] and protons [**(C)** 1-day desiccated samples]. The first lane on the pulsed-field gel electrophoresis correspond to the karyotype of *Saccharomyces cerevisiae*. Second lanes correspond to the control (1,000 hydrated individuals). Third and fourth lanes correspond, respectively, to hydrated or desiccated bdelloids not submitted and submitted to 800 Gy radiation. Other lanes correspond to 1,000 desiccated individuals after 2, 30, 8, 24, 48, and 168 h of rehydration/post exposure. The run parameters are documented under each gel.

The potential repair of DNA fragmentation induced by 800 Gy of X-ray and proton radiation in the somatic cells of (re-)hydrated animals were assessed through a kinetic from 0 to 1-week post exposure (Figures 5A–C). Following the rehydration or irradiation arrest (for hydrated animals), the genomic integrity was characterized by a smear of small DNA fragments (<2,200 kb, average size of 450 kbp) progressively vanishing within 24 h. The large (≥2,200 kb) DNA segments gradually re-appeared in the gel, revealing the presence of an active DNA DSB repair mechanism among *A. vaga* following massive genome breakage. Independently of the radiation type and the hydration state, DNA repair took place but the genomic profile of the control samples was not restored after 48 or 168 h post irradiation: in all tested exposure types, a smear of fragments ranging from 225 to 2,200 kbp was still observed.

## Discussion

This research highlights that lethal doses for desiccated *A. vaga* individuals range between 5,000 to 7,500 Gy using low and high LET radiation (for X-ray and Proton, respectively, see Figure 1). At dose of 5,000 Gy, the survival rate was higher for animals exposed to X-ray (82.7% SD ± 11.4) than to proton radiation (19.1% SD ± 34.1). This is in congruence with the idea that high LET radiation has a higher relative biological effectiveness than low LET radiation (Goodhead, 1999). A similar trend was reported for *A. vaga* samples exposed to high energy Fe particles despite the lack of exposure to doses higher than 3,000 Gy (see Figure 1). Noticeably, doses associated with drop of survival or fertility rates, in both X-ray and proton radiation exposure, were characterized by a higher level of heterogeneity between replicates. We hypothesize that this variability is linked to the uncertainty of the delivered dose. Although this is low (of the order of 1 to 2% of the delivered dose depending on the irradiation system used), it can become non-negligible in view of the total doses delivered in this study. Moreover, the dose received by animals is a Gaussian centered around the average reported dose (Poisson statistic). This is a characteristic specific to the use of broad beam radiation system and is a well-known source of variability in radiobiology. Finally, other uncontrolled biological factors, such as the average age of the exposed population between each assay, may contribute to the variability observed.

The radio-resistance of *A. vaga* individuals was differentially interpreted, in term of magnitude, when focusing on the survival rate or on their capacity to produce viable offspring. In our study, for X-ray for example, a factor of approximately 3 was reported between doses required to sterilize a population or to kill all exposed animals (compare Figures 1, 3: dose required to sterilize 100% *A. vaga* population is between 1,500 and 2,000 Gy X-ray while it is between 5,000 and 7,500 Gy to kill the entire population). At minimal doses required to sterilize *A. vaga* individuals, no decrease of survival rate is observed in comparison with controls (see Figures 1, 3). Animals were sterilized by doses of 750, 2,000 and 1,000 Gy for Protons, X-ray and Fe radiation, respectively (Figure 3). The median sterilizing doses (SD_50_) for the *A. vaga* clone were 1,035 ± 20 Gy, 453 ± 23 Gy and 461 ± 1 Gy for X-ray, protons and iron, respectively (see Supplementary Figure S1). Due to the lack of intermediary dose for Fe particles between 500 and 1,000 Gy, no significant difference was reported between Protons and Fe. Nevertheless, data confirmed a clear difference between low and high LET radiation at the reproductive level. A 2.3 factor was reported when comparing SD_50_ of X-ray versus protons and Fe particles. Indeed, it would have taken twice as much dose deposited in the case of X-rays to sterilize individuals in comparison with protons and iron particles. The precise causes of these differences remain to be investigated but may be informative to the documentation of damage type induced by low and high LET radiation on a radioresistant metazoan.

The observed shift between survival rate and sterilization dose was previously also reported for other metazoan species like tardigrades. *Milnesium tardigradum* survived high doses of gamma radiation in both hydrated (LD_50/48 *h*_ 5,000 ± 1,900 Gy) and anhydrobiotic (LD_50/48 *h*_ 4,400 ± 500 Gy) states, but an irradiation dose >1,000 Gy makes them sterile (Horikawa et al., 2006). Similarly, the tardigrade *Hypsibius dujardini* exposed to gamma radiation presented a LD_50/48 *h*_ for survival estimated at ∼ 4,200 Gy, and doses higher than 100 Gy reduced drastically production and hatchability of laid eggs (Beltran-Pardo et al., 2015). These observations reported for tardigrades and bdelloids, both eutelic, may be attributed to the nature of the cells evaluated through survival or fecundity rate assays. The survival rate in those metazoans is related to non-dividing somatic cells, while fertility data is linked to germinal cells that will ensure cellular multiplication during embryonic development and as consequence are more sensitive to damages induced by radiation (Pagani et al., 1993; Beltran-Pardo et al., 2015). Nevertheless, when compared to other metazoans, the tested bdelloid rotifer and tardigrade species show a very high resistance to ionizing radiation (for examples see Krisko et al., 2012).

For future comparative analysis between radiation sensitive and radioresistant organisms, it may be more relevant to focus on fertility rate. Moreover, in term of evolution, there is no advantage to survive to extreme dose of radiation if no offspring is produced. As expected, the exposure to high doses of ionizing radiation was visualized through PFGE as an increased level of DNA fragmentation in function of the dose (Figure 4). An attempt was made here to discriminate DNA fragmentation induced by low and high LET, as well as the contribution of indirect damages by exposing hydrated *A. vaga* individuals to X-ray. No significant difference was reported between radiation source for comparable dose when focusing on fragment sizes ranging between 225 and 1,600 kbp. Similarly, it was not possible to detect significant differences in DNA fragmentation between samples exposed in hydrated or desiccated state (Figure 4). Nevertheless, absence of differences may be due to the lack of resolution inherent to PFGE focusing on high molecular weight DNA fragments. The resolution range of 225 kbp to 1,600 kbp is hypothesized to miss clustered damages induced by high LET radiation that may result in short DNA fragments. A comparative analysis was performed in order to check the genomic integrity pattern of fragments ranging from 15 to 240 kbp. Using this resolution window, it was possible to observe different DNA migration patterns between X-ray and proton irradiated *A. vaga* individuals (see Figure 4F). The increased smear of short size DNA fragments reported after proton exposure, in comparison to X-ray irradiated samples, may be attributed to the clustered damages generated by proton exposure resulting in the higher relative biological effectiveness of proton radiation vs X-rays. However, these data need to be validated by a systematic approach testing the impact of different particles, with increasing LET and other resolution range. Indeed, fine quantification of short DNA fragments induced by radiation is still challenging (Höglund et al., 2000; Alloni et al., 2013; Barbieri et al., 2019).

The loss of genomic integrity induced by low or high doses of radiation do not prevent the recovery process of *A. vaga*, neither in hydrated nor in desiccated state (Figure 4). Despite the dramatic loss of genomic integrity, *A. vaga* individuals were able to repair, at least partially, DNA damage induced by 800 Gy of X-ray or proton radiation in the somatic cells of (re-)hydrated animals (Figure 5). Indeed the 1,000 *A. vaga* individuals of the PFGE analysis contain only few germline cells per individual (<30) that are probably not recorded here, and only the signal of DNA repair in somatic cells is observed (>900). DNA repair kinetics suggest that most DNA damage induced by radiation are repaired within 24 h independently of the radiation source or on the reproductive capacity (see Figures 2, 3, 5). At first glance, these results suggest that the DNA repair kinetic taking place in somatic cells of irradiated *A. vaga* individuals appeared to be insensitive to the complexity of damage induced by low vs. high LET radiation exposures. The exact mechanism of DNA repair and the potential divergence in mechanism taking place in somatic or germline cells remain mostly unknown. Moreover, consequences of such massive DNA fragmentation, expected to occur also in germline cells, remain to be investigated at genomic scale on offspring originated from irradiated individuals. The genome of rotifer *A. vaga* was previously reported to carry an enriched complement of DNA repair genes compared to other invertebrates models (Flot et al., 2013; Hecox-Lea and Mark Welch, 2018). Genes of both homology-directed and non-homologous end joining (NHEJ) repair pathways are present in numerous copies, some of which likely acquired by horizontal transfers (Hecox-Lea and Mark Welch, 2018). Functional genetics are, however, required to determine the exact nature of their DNA repair process.

It is well known that ionizing radiation damages on biological samples can be generated by two paths of action. The first one, called “direct damage” literally means either the incident radiation itself or secondary particles ionize the target and generate breaks in the molecules. The second one, called “indirect action” refers to the generation of effectors such as reactive oxygen species (ROS), most frequently generated through water radiolysis, due to the incident radiation that, in turn, will generate the damages to cell compounds (Dartnell, 2011). In this line of thought, the contribution of direct and indirect damages remains to be investigated at high doses of radiation. The capacity of bdelloid rotifers to be exposed to ionizing radiation in hydrated and desiccated state constitutes an opportunity to evaluate biological effectiveness played by direct and indirect damages. Here, the impact of X-ray on desiccated and hydrated *A. vaga* was investigated. Within a radiation range of 0 to 1,500 Gy, no significant effects on the survival rate of irradiated *A. vaga* were reported 48 h post-exposition and eventual rehydration (see Figure 2A). Nevertheless, SD_50_ of hydrated individuals appeared to be lower than the one calculated for desiccated individuals. These data may suggest an increased level of damages due to the presence of liquid water (see Figure 2B). However, no significant difference was found when compared fertility rate of both conditions. In contrast, a higher proportion of dead animals, unable to produce eggs was reported 30 days post exposure among *A. vaga* individuals exposed in hydrated state starting 1,000 Gy (see Supplementary Figure S3b, group “d”). This latest result may be interpreted as a harmful impact of X-ray on hydrated animals that appeared to die prematurely in comparison with desiccated and irradiated samples. Indeed, the survival rate was only recorded 2 days post irradiation or rehydration and provides, as consequence, an evaluation of it at a short term. Investigation at different time points should be implemented to confirm the premature dead reported in hydrated samples. Inversely, these data may contribute to support the fact that somatic cells of desiccated bdelloid individuals are less affected by radiation in comparison with hydrated ones. According to the literature, 60 to 70% of damages induced *in vitro* cells by X-ray exposure originate from indirect damages (Komatsu, 2019). However, the comparison of SD_50_ from animals exposed hydrated (SD_50_ = 889 ± 12 Gy) or desiccated (1,125 ± 88 Gy) suggested a contribution of only 21% of indirect damages on *A. vaga* fecundity rate (see Supplementary Figure S1b). The radioresistance of *A. vaga* was previously attributed to the presence of an unusually effective system of anti-oxidant protection, protecting also the DNA repair proteins required to repair the fragmented genome (Krisko et al., 2012). The reduced contribution of indirect damage observed here in hydrated, exposed samples may be linked to the efficiency of the antioxidant protection system present in *A. vaga*. Complementary studies must be addressed to document the biological response of living organisms to low and high LET radiation as well as the contribution of direct and indirect damage using desiccated and hydrated individuals.

## Conclusion

In conclusion, the robustness of bdelloid rotifers against multiple stresses coupled with their desiccation tolerance at any stage of their life cycle prone them to the short list of animals that can be used as model systems to study how life can be adapted and could evolve in extra-terrestrial environments (Jönsson et al., 2008; Vukich et al., 2012; Rabbow et al., 2015). As a reference, this study focused on the bdelloid species *A. vaga*. It remains unknown if *A. vaga*, isolated 40 years ago from the wild and continuously maintained hydrated in laboratory condition, may reflect the overall limit of bdelloid radioresistance. Moreover, it is not excluded that this species presents a lower radioresistant phenotype in comparison with species living in extreme environments and Mars analog sites like Antarctic or Atacama Desert (Robeson et al., 2009; Iakovenko et al., 2015). Further studies are ongoing to decipher the impact of the environment of bdelloid species to their desiccation/radiation tolerance. Finally, the exposure of biological organisms in laboratory remains a simple view of the complex radiation flux found in space environment (Moeller et al., 2017) and experiments ongoing on ISS may reveal more insights into the resistance to cosmic radiation and space environment. Nevertheless, the combination of different type of low and high LET radiations, exposure to UV radiation, variation of temperature may significantly affect the biological effectiveness of unprotected samples ongoing lithopanspermia or “living” on martian-like atmosphere.

## Data Availability Statement

All datasets generated for this study are included in the article/Supplementary Material.

## Author Contributions

BH, LB, CB, and VB maintained the rotifer cultures. BH and LB performed all the experiments with help of CB and VB. Exposures to radiation were performed by BH, VB, CB, and LB under the supervision of A-CH, SL, RM, and AF. The results were analyzed by BH, VC, BR, and SP. The manuscript was written by BH. The manuscript was revised by KV, A-CH, SL, VC, RM, AF, and SP. The project was designed and supervised by BH and KV. The acquisition of funding was done by BH and KV. All authors contributed to the article and approved the submitted version.

## Conflict of Interest

The authors declare that the research was conducted in the absence of any commercial or financial relationships that could be construed as a potential conflict of interest.

## Supplementary Material

FIGURE S1 Evaluation of Sterilizing Dose 50 (SD_50_, Gy) for *A. vaga* individuals exposed to X-ray **(a,b)**, protons **(c)** and Fe **(d)**, overview **(e)**. Each dose was tested in min 3 replicates. For each replicate, 60 individuals were randomly isolated and individually placed in multiwell plates. Reproduction was evaluated by direct observation under binocular 30 days after irradiation and rehydration. All curve fittings were performed with the OriginLab^®^ software (MA, United States) (see section “Materials and Methods”). Two SD_50_ for desiccated *A. vaga* species irradiated with X-ray were calculated. First SD_50_ included all data from 0 to 7,500 Gy X-ray. The second SD*_50_* was calculated by limiting the dose range from 0 to 1,500 Gy X-ray. This last value was used for comparison with SD_50_ of hydrated specimens exposed to X-ray similarly calculated with dose from 0 to 1,500 Gy.

FIGURE S2 Production of sterile eggs or premature dead in desiccated *A. vaga* individuals exposed to **(a)** protons (P +) **(b)** X-ray (Xr) c. Fe. For each replicate, 60 individuals were randomly isolated and individually placed in multi-well plates. Reproduction was evaluated by direct observation under binocular 30 days after irradiation and rehydration. Samples were characterized as “Sterile egg production” when one *A. vaga* individual and minimum one sterile egg was reported. If animals died within a month or were not able to lay eggs, it was annotated as “Dead or no egg production.” When the number of survivors was below 60 individuals, the number of isolated individuals lacking to arrive at this total was automatically associated with the category “Dead or no egg production.” This correction avoids the overweighting of certain values following the drastic reduction of survivors at dose equal or higher than 5,000 Gy (X-ray and proton only). Data were visualized as boxplot (◯ = average; – = median). Letters indicate the significant differences between groups (*P*-value < 0.05; see section “Materials and Methods”).

FIGURE S3 Production of sterile eggs **(a)** or premature dead **(b)** in *A. vaga* individuals exposed to X-ray in desiccated (Red) or hydrated (white) state. Each dose was tested in min 3 replicates. For each replicate, 60 individuals were randomly isolated and individually placed in multiwell plates. Reproduction was evaluated by direct observation under binocular 30 days after irradiation and rehydration. Sample were characterized as “Sterile eggs production” when one *A. vaga* individuals and min one sterile eggs was reported. If animals died within a month or was not able to lay egg, it was annotated as “Dead or no eggs production.” Statistical analysis of the sterile eggs production highlighted only an effect of dose. Related to premature dead data, an interaction effect between dose factor and hydrated/desiccated status was highlighted. Data were visualized as boxplot (◯ = average; – = median). Letters indicate the significant differences between groups (*P*-value < 0.05; see section “Materials and Methods”).

FIGURE S4 Genomic integrity of 1-day desiccated *A. vaga* individuals exposed to 500 and 1,000 Gy with X-rays and 4 MeV Protons, respectively, using short fragments run parameters (15–291 kbp). Lanes 1 and 5 on the pulsed-field gel electrophoresis corresponds to the karyotype of *Saccharomyces cerevisiae*. Lanes 2 and 6 correspond to the control (1,000 hydrated *A. vaga* individuals). Lanes 3 and 7 correspond to 1-day desiccated *A. vaga* individuals exposed, respectively, to 500 and 1,000 Gy of X-ray radiation. Lanes 4 and 8 correspond to 1-day desiccated *A. vaga* individuals exposed, respectively, to 500 and 1,000 Gy of protons 4 MeV radiation. The run parameters are documented under the gel. Samples used for Supplementary Figure 4, were irradiated and prepared independently from samples used for Figure 4.

DATA S1 Survival and fertility data.
