# Supplementary figures and images for: Iron Ladies – How Desiccated Asexual Rotifer Adineta vaga Deal With X-Rays and Heavy Ions?
Source: Front Microbiol. 2020 Jul 31;11:1792. doi: 10.3389/fmicb.2020.01792 (PMC7412981; doi:10.3389/fmicb.2020.01792)

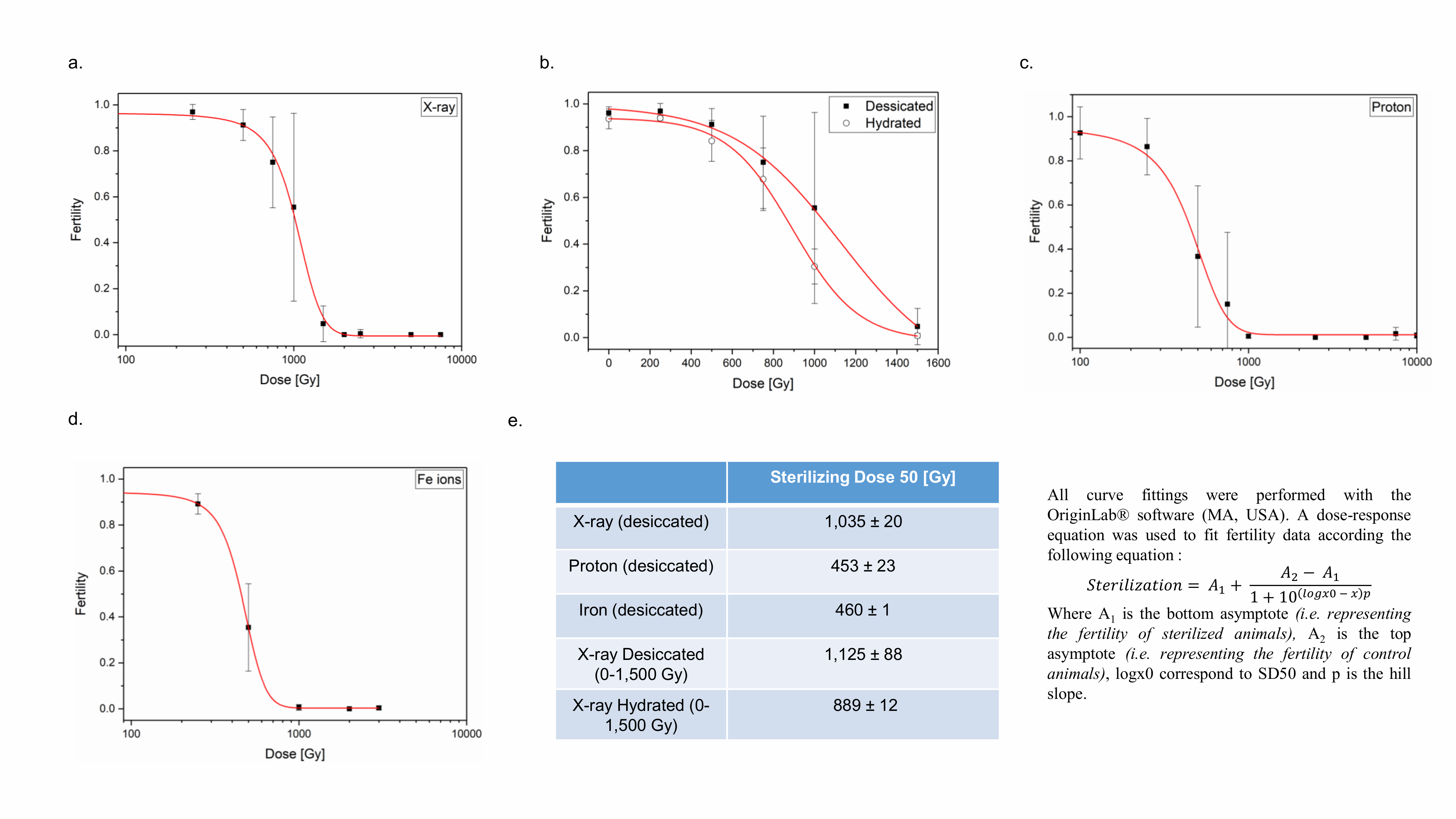

Supplement: FIGURE S1 — Evaluation of Sterilizing Dose 50 (SD50, Gy) for A. vaga individuals exposed to X-ray (a,b), protons (c) and Fe (d), overview (e). Each dose was tested in min 3 replicates. For each replicate, 60 individuals were randomly isolated and individually placed in multiwell plates. Reproduction was evaluated by direct observation under binocular 30 days after irradiation and rehydration. All curve fittings were performed with the OriginLab® software (MA, United States) (see section “Materials and Methods”). Two SD50 for desiccated A. vaga species irradiated with X-ray were calculated. First SD50 included all data from 0 to 7,500 Gy X-ray. The second SD50 was calculated by limiting the dose range from 0 to 1,500 Gy X-ray. This last value was used for comparison with SD50 of hydrated specimens exposed to X-ray similarly calculated with dose from 0 to 1,500 Gy. [file Image_1.TIF]

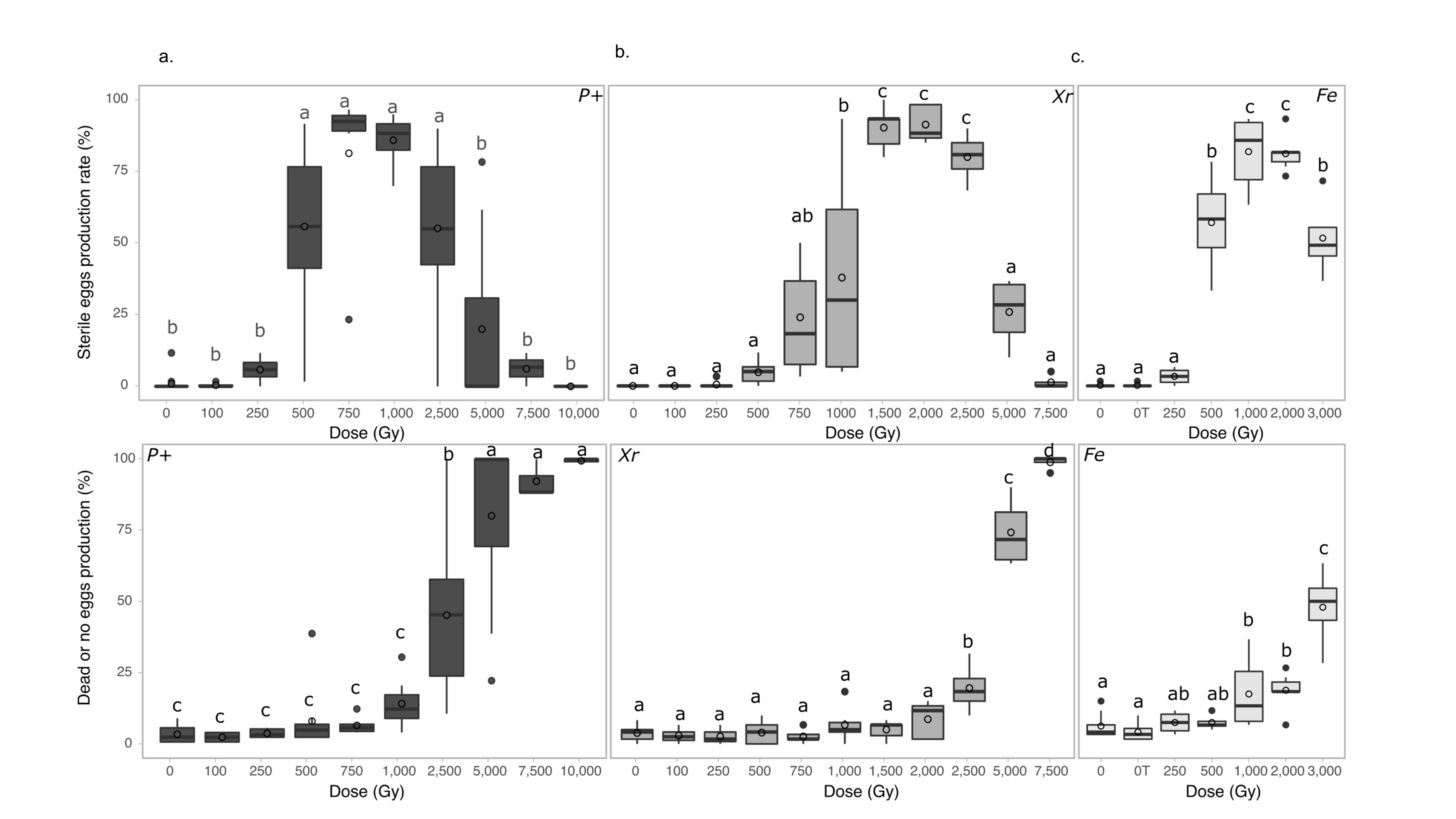

Supplement: FIGURE S2 — Production of sterile eggs or premature dead in desiccated A. vaga individuals exposed to (a) protons (P +) (b) X-ray (Xr) c. Fe. For each replicate, 60 individuals were randomly isolated and individually placed in multi-well plates. Reproduction was evaluated by direct observation under binocular 30 days after irradiation and rehydration. Samples were characterized as “Sterile egg production” when one A. vaga individual and minimum one sterile egg was reported. If animals died within a month or were not able to lay eggs, it was annotated as “Dead or no egg production.” When the number of survivors was below 60 individuals, the number of isolated individuals lacking to arrive at this total was automatically associated with the category “Dead or no egg production.” This correction avoids the overweighting of certain values following the drastic reduction of survivors at dose equal or higher than 5,000 Gy (X-ray and proton only). Data were visualized as boxplot (◯ = average; – = median). Letters indicate the significant differences between groups (P-value < 0.05; see section “Materials and Methods”). [file Image_2.TIF]

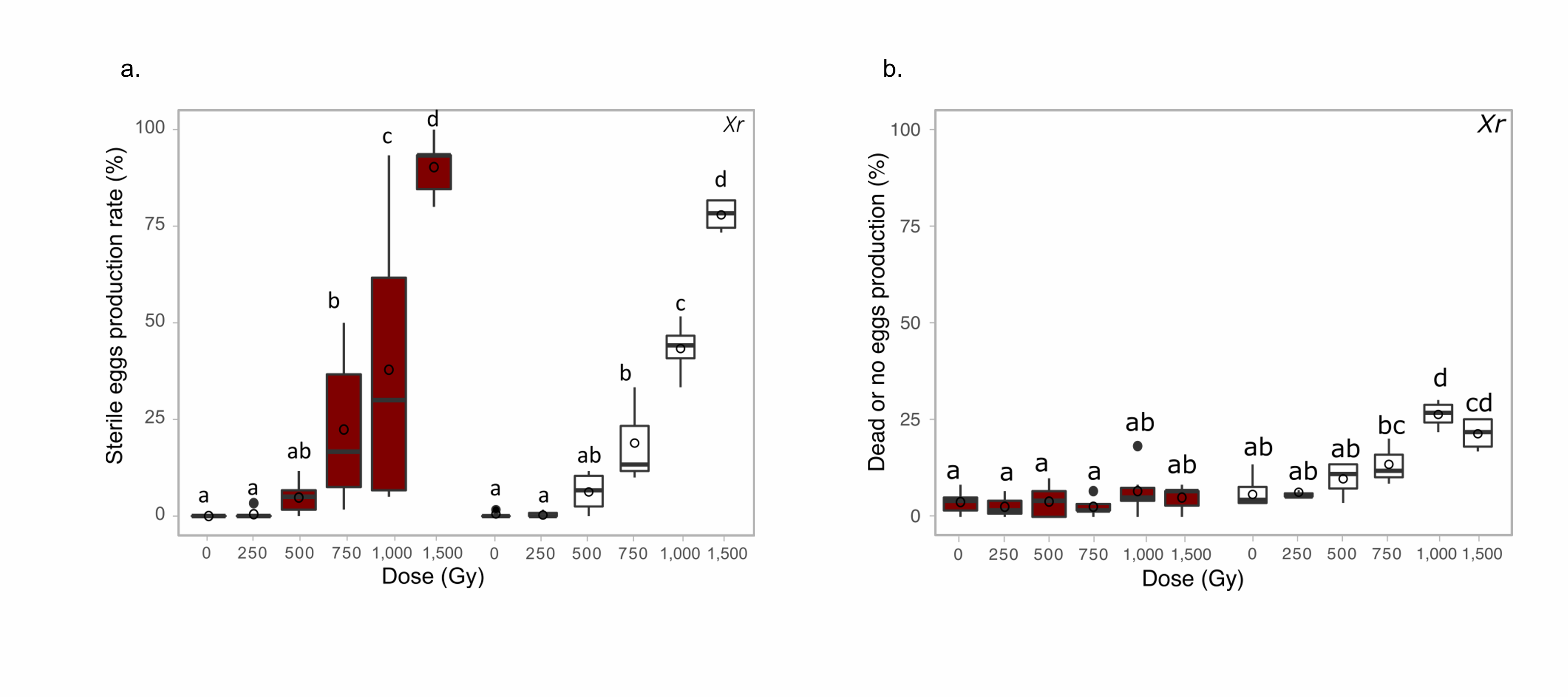

Supplement: FIGURE S3 — Production of sterile eggs (a) or premature dead (b) in A. vaga individuals exposed to X-ray in desiccated (Red) or hydrated (white) state. Each dose was tested in min 3 replicates. For each replicate, 60 individuals were randomly isolated and individually placed in multiwell plates. Reproduction was evaluated by direct observation under binocular 30 days after irradiation and rehydration. Sample were characterized as “Sterile eggs production” when one A. vaga individuals and min one sterile eggs was reported. If animals died within a month or was not able to lay egg, it was annotated as “Dead or no eggs production.” Statistical analysis of the sterile eggs production highlighted only an effect of dose. Related to premature dead data, an interaction effect between dose factor and hydrated/desiccated status was highlighted. Data were visualized as boxplot (◯ = average; – = median). Letters indicate the significant differences between groups (P-value < 0.05; see section “Materials and Methods”). [file Image_3.TIF]

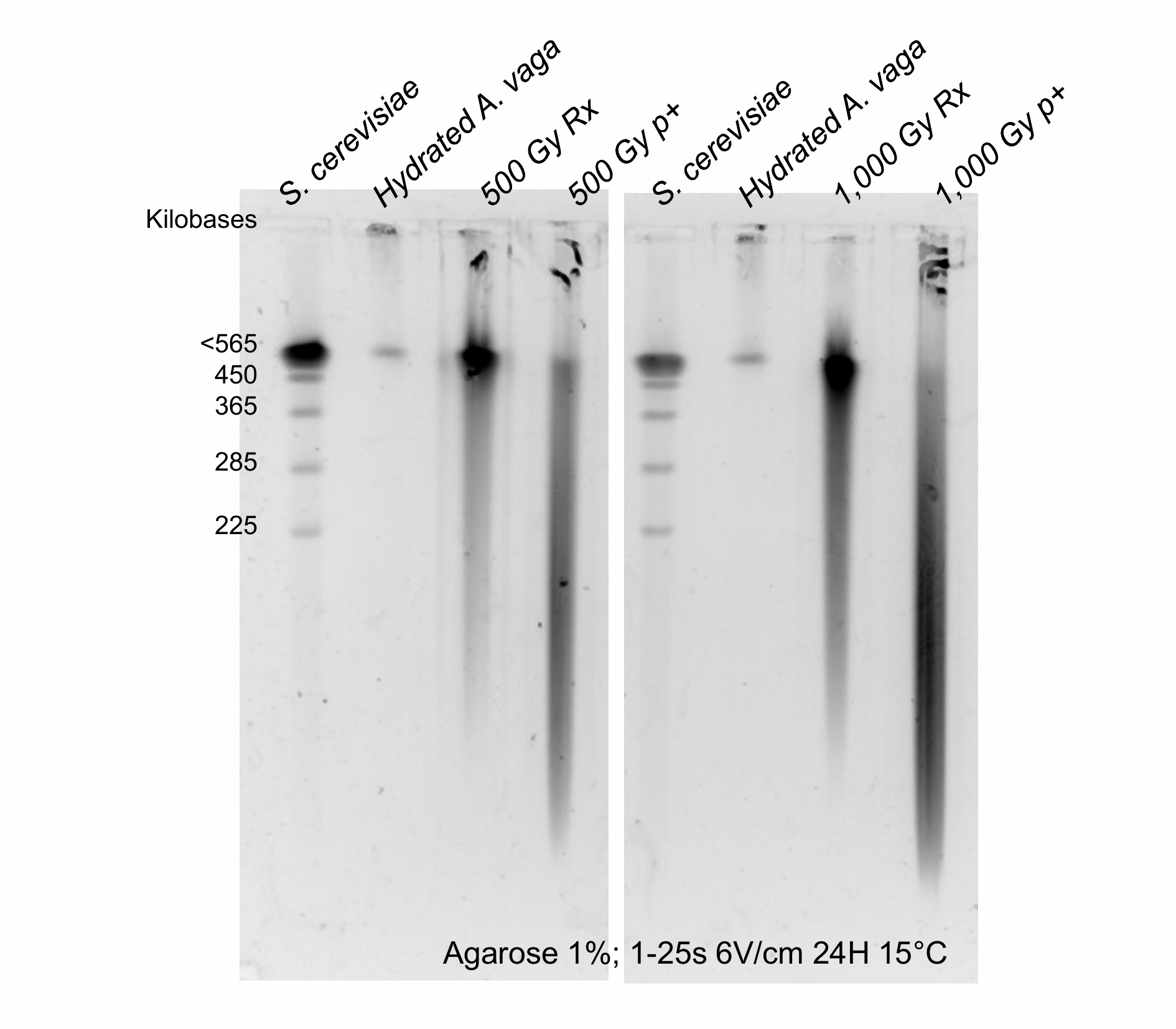

Supplement: FIGURE S4 — Genomic integrity of 1-day desiccated A. vaga individuals exposed to 500 and 1,000 Gy with X-rays and 4 MeV Protons, respectively, using short fragments run parameters (15–291 kbp). Lanes 1 and 5 on the pulsed-field gel electrophoresis corresponds to the karyotype of Saccharomyces cerevisiae. Lanes 2 and 6 correspond to the control (1,000 hydrated A. vaga individuals). Lanes 3 and 7 correspond to 1-day desiccated A. vaga individuals exposed, respectively, to 500 and 1,000 Gy of X-ray radiation. Lanes 4 and 8 correspond to 1-day desiccated A. vaga individuals exposed, respectively, to 500 and 1,000 Gy of protons 4 MeV radiation. The run parameters are documented under the gel. Samples used for Supplementary Figure 4, were irradiated and prepared independently from samples used for Figure 4. [file Image_4.TIF]
